# Foxtail millet [*Setaria italica* (L.) P. Beauv.] grown under nitrogen deficiency exhibits a lower folate contents

**DOI:** 10.3389/fnut.2023.1035739

**Published:** 2023-01-18

**Authors:** Yuan Wang, Jin-song Wang, Er-wei Dong, Qiu-xia Liu, Li-ge Wang, Er-ying Chen, Xiao-yan Jiao, Xian-min Diao

**Affiliations:** ^1^College of Resources and Environment, Shanxi Agricultural University, Taiyuan, Shanxi, China; ^2^Institute of Crop Research, Shandong Academy of Agricultural Sciences, Jinan, China; ^3^Institute of Crop Sciences, Chinese Academy of Agricultural Sciences, Beijing, China

**Keywords:** foxtail millet, cultivars, folates, nitrogen deficiency, plant NPK accumulation, nitrogen recovery efficiency

## Abstract

Foxtail millet [*Setaria italica* (L.) P. Beauv.], as a rich source of folates, has been cultivated on arid infertile lands, for which N deficiency is one of the major issues. Growing environments might have a significant influence on cereal folate levels. However, little is known whether N deficiency modulates cereal folate levels. In order to obtain enriched folate foxtail millet production in nutrient-poor soil, we conducted a study investigating the content of folate derivatives of 29 diverse foxtail millet cultivars under two N regimes (0 and 150 kg N ha^−1^) for 2 years to explore folate potential grown under low N. The contents of total folate and most derivatives were reduced by N deficiency. The effect on total folate content caused by N was stronger than cultivar genotype did. Folate content of enriched folate cultivars was prone to be reduced by N deficiency. Structural equation models (SEMs) revealed that N fertilization had a positive indirect effect on grain folate content through influencing plant N and K accumulation. Collectively, the results indicate much more attention should be paid to N management when foxtail millet is cultivated in infertile soil, to improve foxtail millet folate contents.

## 1. Introduction

Folates (vitamin B9), consisting of tetrahydrofolate (THF) and its derivatives, are essential for nucleotide synthesis and cofactors in one-carbon units. Humans lack the capability of *de novo* folate biosynthesis (1). For this reason, folates are essential micronutrients, and must be supplied through balanced diet. Folate efficiently prevents the neural tube defects in the developing fetus, cardiovascular disease, and stroke, and folate deficiency may lead to megaloblastic anemia (2, 3). It has been reported that the prevalence of folate deficiency in women of reproductive age is more than 40% in most countries, resulting in approximately 300,000 newborns with neural tube defects per year. To prevent neural tube defects, the US Public Health Service has recommended that all women capable of becoming pregnant consume 400 μg per day of folic acid (4). Maruvada et al. (5) reported that food fortification and medical supplementation are suggested as complementary ways to alleviate folate deficiency. However, folic acid supplementation may lead to adverse effects of elevated folate status, such as potential cancers. Thus, food fortification is a cost-effective and efficient alternative. Endogenous folate in wholegrain cereals is readily bioavailable and may improve folate status (6).

Genotypes and environment significantly influence the folate content in cereal. For instance, total folate content among wheat and soybean genotypes shows a remarked variation (7, 8). Moreover, most of the folate in cereal products also differs markedly according to the growing conditions (climate, soil type, weather conditions, etc.) (9). Therefore, genotypes with high folate by careful selection and the means to enhance natural folate contents by improving environmental factors need to be studied.

Nitrogen (N) is an essential, often limiting, factor in plant growth and grain development (10). Upon N limitation, plants develop physiological alterations, including folate synthesis processes (11). According to Jiang et al. (12), N deficiency decreased the expression of most of the genes involved in folate synthesis and C1 units in *Arabidopsis* seedlings. Furthermore, folates are assembled from pterin, ρ-aminobenzoate, and glutamate precursors. Most folates are conjugated to a c-linked polyglutamyl tail of up to eight residues. These polyglutamyl tails may help to protect folates from oxidative breakdown, and folates tend to be stabilized by polyglutamylation (13). In *Arabidopsis*, low N typically results in significant decreases in glutamine and asparagines (14) and might exhibit a less conjugation of polyglutamylated folates. Folate levels correlate positively with polyglutamate tail length (15). These observations indicate that N limitation in the soil might have a detrimental effect on folate levels of grain produced.

Foxtail millet [*Setaria italica* (L.) P. Beauv.], as the second millet in terms of worldwide production after pearl millet (16), has valuable nutritional and medicinal properties (17). It comprises a wide range of health-benefiting components, including phenolic (18), protein hydrolysates (19), carotenoids (20), polysaccharides (21), and other antioxidants, which have also been shown to possess several health benefits like prevention of cancer, hypoglycemic, and hypolipidemic effects (17). These functional components make foxtail millet as an unique nutritional crop among the cereal categories. A few of previous studies revealed that folate contents in foxtail millet were much higher than that in other cereals such as rice, wheat, and maize (22, 23). While several studies have investigated the changes of both folate metabolite content and the expression patterns of folate metabolite-related genes in foxtail millet grain during it developed (24), fewer evaluation concerning the effect of a specific environmental factor on cereal crops folate levels has been carried out, especially for the effect of N deficiency on foxtail millet. Foxtail millet has been cultivated primarily on infertile lands in arid and semi-arid areas of Asia. It is prone to adapt to adverse soils than most other crops (25). Being sessile in nutrient-poor soil, foxtail millet encounters some environmental challenges while obtaining the nutrients necessary for development and biomass production, especially in N deficiency. Therefore, the variation in foxtail millet folate contents under N-deficiency could be used to elucidate foxtail millet folate potential grown in nutrient-poor soil and reveal the effects of agronomic fertilization measures on grain folate content.

In order to survey the natural variation of folate contents among foxtail millet cultivars and their folate potential levels under nutrient-poor soil in China, a total of 29 Chinese foxtail millet cultivars were grown with or without N fertilization in 2020 and 2021 and were analyzed for folate contents by using HPLC-MS/MS. The aims of this study were: (1) to investigate the folate content variation of the leading Chinese foxtail millet cultivars; (2) to assess the effect of N deficiency on folate content; (3) to evaluate the association of foxtail millet folate levels with nutrient accumulation, which was affected by N regimes. The findings of this study would provide essential information for folate improvement in foxtail millet and other cereals. In particular, much attention should be paid to fertilization management when cultivating elite folate cultivars, especially in infertile soil conditions.

## 2. Materials and methods

### 2.1. Plant materials and design of experiment

**Figure 1:**
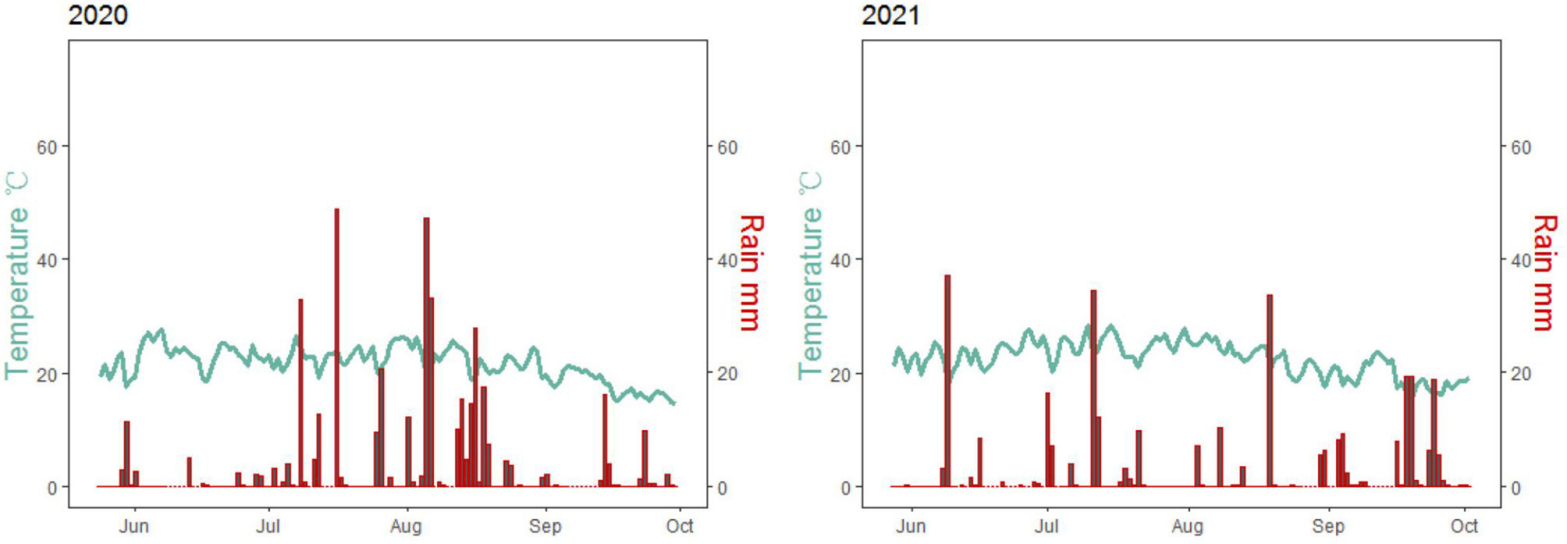
Average daily temperature and rainfall during growing seasons in 2020 and 2021.

Field experiments were conducted at the Dongyang Experimental Station of Shanxi Agricultural University, Shanxi, China (37°33′21″N, 112°40′2″E) in 2020 and 2021. This site generally experiences a temperate continental climate with a mean annual air temperature of 9.7°C and 440.7 mm of rainfall. The precipitation during the foxtail millet growth period was 411.8 mm and 310.8 mm in 2020 and 2021, respectively (Figure 1). This site possesses sandy loam soil with the pH of 8.45 and contained 0.93 g kg^−1^ total N, 6.87 mg kg^−1^ Olsen-P (P), 138.2 mg kg^−1^ available potassium (K), and 20.33 g kg^−1^ organic matter.

The field experiment was a twenty-nine by two [most widely used and representative twenty-nine (29) foxtail millet cultivars and two levels of N rates] factorial design with 58 combinations, in which each combination had three plots as replicates. Two N rates were included 0 kg N ha^−1^ application (N-) and 150 kg N ha^−1^ application (N+). Nitrogen was supplied as slow-release urea (46%-N) before sowing. For all treatments, 75 kg ha^−1^ P_2_O_5_ and 75 kg ha^−1^ K_2_O were applied before sowing as well. Each plot area was 30 m^2^ (5 × 6 m). Plants on the borders of the plots were treated for protection and not harvested. The information of sowing date, plant density, plant height with N applied, and harvest date (the mature stage of the selected cultivars) of every selected cultivar is shown in Supplementary Table 1. At the three-leaf stage, seedlings were thinned to the suitable density of every selected cultivar. A total of 120 mm of water was applied through irrigation, half applied before fertilizer application and the rest irrigated before heading. Weeds, diseases, and insect pests were controlled adequately; No factor other than the N level limited growth.

### 2.2. Sampling

When panicles and grains were fully matured and plant individuals were harvested from 20 m^2^ of each plot, then panicle weight, straw weight, and grain weight were estimated, and the thousand-grain weight was determined. The total weight of panicles and straw was used to compute aboveground biomass. Some representative plant samples (grain, panicle, and straw) were used to collect tissue for N, P, and K analysis after drying at 75°C to maintain a constant weight. After being weighted, the samples were finely grounded to pass through a 250 μm sieve. Another portion of representative grains from each cultivar was stored in a paper bag after threshing on time. About 5 g grains were manually ground to fine powder below 4°C, and the powder was completely filtered through the screen mesh (100 μm sieve). Then powder was temporarily stored at −80°C until further analyses of the folate levels.

### 2.3. Determination of N, P, and K accumulation in plant tissues

According to the micro-Kjeldahl method, N content in plant tissues was determined with automatic Kjeldahl Apparatus (FOSS-8400, Sweden) (26). Furthermore, about 0.5 g ground sample was digested by microwave-assisted acid digestion (27), followed by total P and K analysis using a vanadium-molybdenum yellow colorimetric method and flame photometer (28), respectively. Grain N, P, and K accumulation per hectare were calculated as grain N, P, or K concentration times grain dry weight per hectare. Total NPK accumulation per hectare during harvest was calculated as the product of NPK concentration and yield of above-ground parts of the foxtail millet plant on a dry matter basis per hectare. Nitrogen recovery efficiency (NRE) was estimated based on the difference in the total plant N uptake between two N treatments in 2 years divided by the total N rate applied in 2 years.

### 2.4. Extraction of folate

#### 2.4.1. Chemical compounds

The folate standards: 10-formyl-folic acid (10–CHO-PteGlu; 10–CHO–FA), 5,10-methenyl-5,6,7,8-tetrahydrofolate (5,10–CH=H_4_PteGlu; 5,10–CH=THF), 5-formyl-tetrahydrofolate (5–CHO-H_4_PteGlu; 5–CHO–THF), 5-methyl-tetrahydrofolate (5–CH_3_-H_4_PteGlu; 5–CH_3_-THF), dihydrofolate (H_2_PteGlu; DHF), folic acid (PteGlu; FA), tetrahydrofolate (H_4_PteGlu; THF), and methotrexate (MTX) were purchased from Schircks Laboratories (Jona, Switzerland) and MeFox, an oxidation product of 5–CH_3_-THF, was obtained from Toronto Research Chemicals (Toronto, Canada). Sodium phosphate dibasic (Na_2_HPO_4_), sodium phosphate monobasic (NaH_2_PO_4_), sodium ascorbate, and β-mercaptoethanol were obtained from the Sigma-Aldrich Chemical. The endogenous folates in rat serum were removed by incubation with one-tenth (w/w) of activated charcoal for 1 h on ice, followed by centrifuging at 13,000 rpm at 4°C for 30 min (Sigma 3K15, Osterode am Harz, Germany), and the supernatant was used for the following incubation experiment. Acetonitrile and formic acid (LC-MS grade) were obtained from Fisher Scientific (Geel, Belgium). The HPLC analytical column (Kromasil 100-5 C18, 2.1 × 50 mm, 2.5 μM particle size) was purchased from Akzo Nobel (Stockholm, Sweden), and an Agilent SB-C18 pre-column (2.1 × 5 mm, 2.7 μM particle size) was acquired from Agilent Technologies (California, USA).

#### 2.4.2. Folate extraction and deglutamylation

Both extraction and measurement of folates were conducted as previously described for wheat (7), with slight modifications. Briefly, folate extraction was performed under subdued light to minimize degradation. The moisture content of each sample was measured after oven overnight at 75°C. Another 50 mg of powder was transferred into a 1.5 mL screw-cap tube (Axygen, ST-150) and 1 mL of freshly prepared extraction solution [5 mM phosphate buffer, pH 7.2; 0.5% sodium ascorbate; and 0.2% β-mercaptoethanol] was added.

Post homogenization, the mixture was immediately boiled in a water bath for 10 min using an electromagnetic oven, cooled on ice for 10 min, then centrifuged for 10 min at 13,000 rpm at 4°C. The supernatant (0.5 mL) was transferred to a fresh tube. Then 35 mL of rat serum was added, and the polyglutamylated tails were deconjugated by incubation at 37°C for 4 h. Consequently, the samples were boiled for 10 min, cooled on ice for 10 min, and centrifuged at 13,000 rpm at 4°C for 10 min. The supernatant was moved to 3 kDa ultra-filtration tubes (Millipore) for cleanup and centrifuged at 13,000 rpm at 4°C for 25 min. Finally, the resulting solution was collected and 100 μL was transferred into new tubes for direct folate detection. The remaining solution was stored at −80°C. Each sample was repeated three times.

### 2.5. Folate standard solutions

The chemical powder of folate standards was dissolved as 0.1 mg/mL in a solution of 20 mM ammonium acetate in methanol and water (1:1, v/v) containing 1% (w/v) L-ascorbic acid, and 0.5% (v/v) β-mercaptoethanol (pH 6.2). 5,10–CH=THF standard was prepared in a pH 4.5 buffer. All standard solutions were kept at −80°C. For spiking and calibration, working solutions were diluted with folate extraction buffer, which was made fresh and used the same day.

### 2.6. Folate determination by HPLC–MS/MS

Chromatographic studies were carried out at a flow rate of 0.30 mL/min using an Agilent 1260 HPLC system (Palo Alto, CA) with an Akzo Nobel analytical column (Kromasil 100-5 C18, 50^*^2.1 mm). The injection volume was 15.0 μL. The injector and column oven temperature were separately maintained at 4 and 25°C, respectively. The mobile phases were 0.1% (v/v) formic acid in water (phase A) and 0.1% (v/v) formic acid in acetonitrile (phase B). The gradient program ran for a total of 19 min. The proportion of mobile phase B increased linearly from 5 to 9% over 2 min. In the following 6 min, phase B increased to 9.6% and then sharply increased to 20% in over 0.2 min. After holding steady at 20% for 3 min, the proportion of phase B decreased to 5% in 0.2 min, followed by a subsequent equilibration.

An Agilent 6420 triple, quadruple tandem MS coupled with an ESI (electron spray ionization) interface was used for mass analyses and quantification of target analytes. The mass spectrometer was used in positive ion mode. The parameters were optimized for the target analytes with a gas temperature of 350°C, drying gas flow at 11 L/min, nebulizer pressure at 35 psi, and capillary voltage at 3500 V (+). The parameters for folate standards were m/z 456–412, 30 eV for 5,10–CH=THF, m/z 460–313, 20 eV for 5–CH3–THF, m/z 474–327, 20 eV for 5–CHO–THF, m/z 446–299, 20 eV for THF, and m/z 455–308, 30 eV for the internal control MTX. System operation and data acquisition were performed with MassHunter software. The sum of contents of different folate derivatives represents as the total folate levels. The seven folate derivatives chromatograms of representative cultivar, Jingu21, under two N treatments were exhibited in Supplementary Figure 1.

### 2.7. Method validation

The precision, linearity, sensitivity, recovery and matrix effect were evaluated for method validation, for which a series of dilution of folate standards was prepared in the blank foxtail millet solutions. To prepare a blank foxtail millet matrix, 1 g fine sample of Jingu21 was mixed with 20 mL phosphate buffer without sodium ascorbate and β-mercaptoethanol. The mixture was boiled for 1 h, then exposed to direct sunlight to degrade endogenous folates. Ten percent activated charcoal was added to the supernatant and was incubated with shaking for 1 h, and then centrifuged at 13,000 rpm for 30 min at 4°C. The supernatant was filtered through 3KDa MWCO ultra-filtration tubes (29).

Precision was assessed based on the relative standard deviation (RSD) of peak areas of Jingu 21 (*n* = 6) on the same and different days. Intra-day repeatability and inter-day repeatability was evaluated by running samples on three different days (Supplementary Table 2). The calibration curves was evaluated by preparing nine-point (1, 2, 5, 10, 50, 100, 200, 500, 1,000 ng/mL) blank foxtail millet solution for MTX and each folate derivative (*n* = 3). The linearity and correlation coefficients were calculated by plotting the peak area at different concentrations. Data analyses were performed with MassHunter software. The correlation coefficients (*R*^2^) for all these folates were approximately > 0.99 in the folate derivatives matrices (Supplementary Table 2). Sensitivity was evaluated by determining the limit of detection (LOD) and limit of quantification (LOQ). The LOD and LOQ values of the amount of analyte were estimated for spiked samples on signal/noise ratios of 3:1 and 10:1, respectively. The LOD and LOQ of the established method in folate derivatives and MTX were in the range of 0.05–0.25 μg per 100 g and 0.16–0.85 μg per 100 g, respectively (Supplementary Table 2). Recovery and matrix effect were calculated as described by Matuszewski et al. (30). The recoveries and matrix effects were illuminated in Supplementary Table 2.

### 2.8. Statistical analysis

The data of folate derivatives in cereal samples were shown as means ± SE of three biological replicates in micrograms per 100 g of grains, with the exception of the contents of folate derivatives under different NRE levels (**Table 2**). Data were exposed to the analysis of variance (ANOVA) with agricolae package and boxplots with the ggplot 2 package in R 4.0.1 (R Foundation for TUNA Team, Tsinghua University, China, Beijing). Pearson correlation was performed on the R statistical software (Performance Analytics and ggplot2).

Structural equation models (SEMs) were established to test the direct and indirect effects of N regimes, plant NPK accumulation, and grain NPK accumulation on folate levels (5–CHO–THF, 5–CH_3_-THF, THF, 10–CHO–FA, and 5,10–CH=THF) in foxtail millets, in which this composite variable did not alter the underlying SEM model. A direct path from N treatment to plant NPK accumulation was used to account for the effects of nutrient utilization efficiency on the plant. A direct path from plant NPK accumulation to grain NPK accumulation was used to account for the effects of crop growth and nutrient distribution on grain nutrient uptake. Folate level was assumed to be affected by all other components both directly and indirectly. We started with a priori models that included all plausible pathways between these factors. Subsequently, the significance of each path coefficient was tested by calculating its critical ratio (*P* < 0.05). The SEMs was performed using the software Amos Graphics v22 (IBM Corp., Armonk, NY, USA). The capacity of SEM to separate the direct and indirect effects of a variable on dependent variables is considered one of the most important advantages of SEM (31). The overall fit of the final model was evaluated with the goodness-of-fit index (GFI), Bentler comparative fit index (CFI), Chi-square test, and root mean square error of approximation (RMSEA) (32).

## 3. Results and discussion

### 3.1. Effects of cultivars and N deficiency on total folate contents in foxtail millets

**Figure 2:**
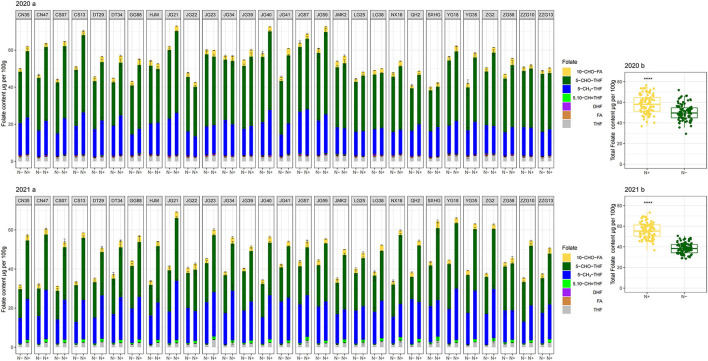
Folate profiling in folate derivatives of the selected cultivars (2020a in 2020; 2021a in 2021) and the total folate of all cultivars (2020b in 2020; 2021b in 2021) grown with or without N fertilization. CN35, Changnong35; CN47, Changnong47; CS07, Changsheng07; CS13, Changsheng13; DT29, Datong29; DT34, Datong34; GG88, Gonggu88; HJM, Huangjinmiao; JG21, Jingu21; JG22, Jigu22; JG23, Jiugu23; JG34, Jingu34; JG39, Jigu39; JG40, Jingu40; JG41, Jigu41; JG57, Jingu57; JG59, Jingu59; JMK2, JinmiaoK2; LG25, Longgu25; LG38, Longgu38; NX18, Nenxuan18; QH2, Qinhuang2; SXHG, shanxihonggu; YG18, Yugu18; YG35, Yugu35; ZG2, Zhonggu2; ZG58, Zhaogu58; ZZG10, Zhangzagu10; ZZG13, Zhangzagu13; N–, N deficiency; N+, N applied treatment. Error bars represent the standard error of folate derivatives. ****Indicate significant differences at 0.0001.

When N (N+) was supplied, the folate levels of 29 foxtail millet cultivars ranged from 42.36 ± 2.91 to 72.89 ± 0.50 μg per 100 g grains and 42.84 ± 1.60 to 69.20 ± 2.13 μg per 100 g grains for 2020 and 2021, respectively (Figure 2 2020b and 2021b). Among the 29 foxtail millet cultivars, only one cultivar (Jingu 21) had mean total folate content of more than 70 μg per 100 g grains for 2 years. About 31% of cultivars (Jingu40, Changsheng13, Yugu18, Jingu59, Zhonggu2, Changnong47, Jingu57, Jiugu23, and Yugu35) had mean total folate content that ranged from 60–70 μg per 100 g for 2 years. A total of 17 cultivars (Changnong35, Changsheng07, Jigu41, Nenxuan18, Jingu34, Gonggu88, Jigu39, Datong34, Datong29, Shanxihonggu, JinmiaoK2, Huangjinmiao, Zhangzagu10, Qinhuang2, Longgu38, Zhaogu58, and Zhangzagu13) fell in the 50-60 μg per 100 g range, and two cultivars (Longgu25, and Jigu22) measured less than 50 μg per 100 g, but over 42 μg per 100 g grains for 2 years (Figure 2 2020a and 2021a). The average folate content of the richest cultivar (e.g. Jingu21) was 1.7-fold higher than that of the poorest cultivar (e.g. Jigu22) under N-sufficient growth conditions. Thus there were obvious differences in folate content among cultivars. Averagely, the total folate content of 2 years for 29 cultivars ranged from 42.59 to 71.03 μg per 100 g. Relative to the folates content in wheat (38–43 μg per 100 g) and rice (6–8 μg per 100 g) reported by Bekaert et al. (23), our study further demonstrated that the foxtail millet cultivars rich in folates could serve as health-benefiting cereals. The total folate contents of the cultivars evaluated in the present study fall within the range of contents reported in previous studies (40.35–111.1 μg per 100 g) (24).

Two-way analysis of variance showed that both two N regimes and cultivars produced highly significant variation in the total folate contents in 2 years, with N factors affecting folates contents much more strongly than that of the cultivar genotypes (mean squares of 2685 and 271 in 2020; mean squares of 13032 and 91 in 2021, respectively) (Supplementary Table 3). Among the 29 foxtail millet cultivars, the folate levels ranged from 40.02 ± 1.74 to 63.72 ± 5.58 μg per 100 g grains when N was deficient in 2020. And the year of 2021, low-N stress affected total folate content much more than that of 2020. During the year of 2021, the folate levels ranged from 31.12 ± 1.57 to 45.25 ± 2.07 μg per 100 g grains under low-N (Figure 2 2020b and 2021b). This demonstrated that increased N stress diminishes folate content seriously. Nitrogen deficiency significantly reduced the total folate contents in the foxtail millet. This may be due to that, typically low N results in large decreases in glutamine and asparagines (14), and exhibits a less conjugation of polyglutamylated folates. These polyglutamyl tails may help to protect folates from oxidative breakdown. As a result, folates tend to be stabilized by polyglutamylation (13). Folate levels correlate positively with polyglutamate tail length (15). As previously stated, folates play an important role in various metabolic processes, including amino acid synthesis (33). This indicates that N status in plant tissues might strongly link with folates. However, further studies will be necessary to explore the mechanism of detrimental effects on folates induced by low-N stress.

The responses of total folate content of 29 cultivars to N deficiency differed significantly. Some foxtail millet cultivars (such as Jingu21, Jingu59, Jingu57, and so on) were found to have relatively high folate contents compared with others for each N regime, but obvious differences in folate content have appeared between two N treatments for these cultivars, in which the reduction ratio of Jingu21 was 47% in 2021 (Figure 2 2020a and 2021a). It indicates that much attention should be paid to N fertilization management when elite folate-rich cultivars are cultivated, especially in infertile soil conditions. Sufficient N should be supplied to ensure elite folate-rich foxtail millet's potential. There were no significant differences in total folate content of Zhaogu58 and Jigu22 detected between two N treatments for both years; whereas both had a relatively low folate content among 29 cultivars (Figure 2 2020a and 2021a). Further, low N stress decreased the difference of folates level among 29 cultivars. Thus, high folate cultivar selection should be conducted in N sufficient conditions in practice.

### 3.2. Effects of N deficiency on folate derivatives contents in foxtail millets

Folates and their derivatives occur as polyglutamates in nature. The extreme low concentrations of folate derivatives limit the applicability of the separation technique and emphasize the demand for sensitive detection techniques (34). Owing to the sensitive detection techniques, the folate levels were analyzed by HPLC- MS/MS in this study, which is supported by Upadhyaya et al. (35). The content of seven folate forms, including 5–CHO–THF, 5–CH_3_-THF, THF, 5,10–CH=THF, 10–CHO–FA, DHF and FA were detected in present study. Other folate forms failed to be detected due to their trace quantities (Figure 2 2020 and 2021).

**Figure 3:**
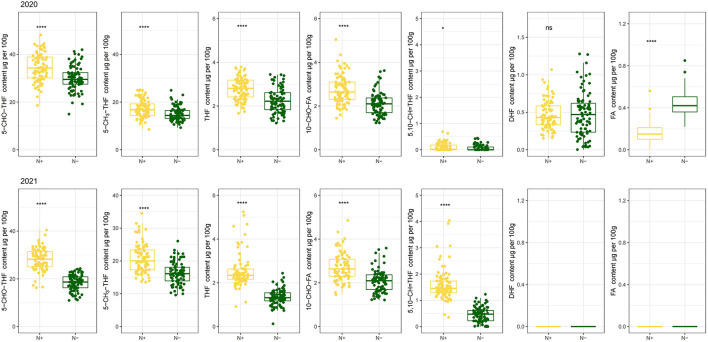
The distribution of each folate derivative 5–CHO–THF, 5–CH_3_-THF, THF, 10–CHO–FA, 5, 10–CH=THF, DHF and FA grown with or without N fertilization. N–, N deficiency; N+, N applied treatment. *, **** and ns indicate significant differences at 0.05, 0.0001 and no significant difference, respectively.

The distribution pattern of the seven derivatives showed that 5–CHO–THF and 5–CH_3_-THF were the predominant folate forms in the foxtail millet cultivars. They contributed to quantifying more than 85% of the total folates, in which 5–CHO–THF contributed more than 45%. Meanwhile, total content of 10–CHO–FA and THF accounted for about 5% of total folate. While FA and DHF were the least abundant folate vitamers, collectively contributing about 1% of total folate in 2020, and failed to be detected because of their trace quantities in 2021 (Figure 2 2021b and Figure 3 2021). Studies on folate vitamer distribution in foxtail millet are scanty. This research indicated that 5–CHO–THF, was the most stable derivative, and the major folate form in foxtail millet; which is also consistent with a previous study in wheat (9, 36), where the content of folate decreases in the following order: 5–CHO–THF > 5–CH_3_-THF > THF (37). This might be related to 5–CHO–THF acting as a storage form of one-carbon groups in seeds (38, 39). Furthermore, 5–CH_3_-THF is one of the folate forms with great bioavailability for animals (40). Therefore, the foxtail millet cultivars with higher 5–CH_3_-THF content can be considered the preferred folate form of food cereals. The present study showed that the highest amounts of 5–CH_3_-THF were 30.29 μg per 100 g grains (Jingu 21 in 2021) when N was supplied (Figure 2 2021a).

Previous studies demonstrated that crop genotypes differ in NRE in the same N-supply environments (41). As mentioned above (Figures 2, 3), N modulated folates content of foxtail millet. Consequently, exploring the relationship between folate levels and N use efficiency is essential. Nitrogen recovery efficiency is an important parameter for N use efficiency. Indeed, Pearson correlation coefficients demonstrated a strong positive relationship between NRE and 5–CH_3_-THF (*P* < 0.01), 5–CHO–THF (*P* < 0.01) or total folate (*P* < 0.01) under sufficient N conditions in two successive years, respectively (Table 1). When the cultivars had more than 45% NRE, its contents of 5–CHO–THF, 5–CH_3_-THF and total folate were obviously high. Their average contents were increased by 17, 19, and 16%, respectively, compared to the contents of cultivars with a low NRE level (< 20%) (Table 2). This probably indicated the foxtail millet cultivars with higher levels of 5–CHO–THF, 5–CH_3_-THF, and total folate might have the ability to absorb more N in soils at the same N application rates. Such high N-use efficiency foxtail millet genotypes probably have the relative high folates potential. Therefore high folate content cultivars might be selected from high N-use efficient genotypes.

**Table 1:** Correlation between NRE and different folate derivatives with N application.

**Correlations with**	**Folate derivatives**							
	**5–CHO–THF**	**5–CH_3_-THF**	**THF**	**10–CHO–FA**	**5,10–CH=THF**	**DHF**	**FA**	**Total**
**NRE**	0.391^**^	0.280^**^	0.032	−0.097	−0.019	0.118	0.106	0.384^**^

** Indicate significant differences at 0.01.

**Table 2:** The contents of different folate derivatives with different NRE levels of cultivars.

**NRE (%)**	**Folate content (**μ**g/100 g)**							
	**5–CHO–THF**	**5–CH_3_-THF**	**THF**	**10–CHO–FA**	**5,10–CH=THF**	**DHF**	**FA**	**Total**
< 20	30.13 ± 2.87a	17.59 ± 2.63a	2.63 ± 0.62a	2.62 ± 0.38ab	0.80 ± 0.38a	0.22 ± 0.09a	0.07 ± 0.04a	54.06 ± 4.30a
20-30	30.30 ± 3.60a	18.71 ± 3.51a	2.67 ± 0.37a	2.86 ± 0.56b	0.93 ± 0.34a	0.23 ± 0.10a	0.08 ± 0.05a	55.80 ± 5.31ab
30-45	31.87 ± 4.20a	19.70 ± 3.82ab	2.65 ± 0.46a	2.78 ± 0.32b	0.79 ± 0.23a	0.24 ± 0.08a	0.08 ± 0.06a	58.11 ± 7.87b
>45	35.35 ± 2.46b	20.94 ± 1.39b	2.74 ± 0.22a	2.48 ± 0.27a	0.82 ± 0.16a	0.25 ± 0.12a	0.10 ± 0.05a	62.67 ± 3.38c

Data are means of three replications ± standard deviation (Mean, SD). The different lowercase letters indicate statistical differences at P < 0.05.

Low-N stress had a great negative effect on 5–CHO–THF, 5–CH_3_-THF, THF, 10–CHO–FA and 5,10–CH=THF for 2 years. Compared with the values when N was applied, N stress resulted in diminished the contents of 5–CHO–THF, by 13.56% and 34.67% in 2020 and 2021, and the content of 5–CH_3_-THF by 12.71% and 22.07% in 2020 and 2021, respectively. Also, the decreased contents of THF, 10–CHO–FA and 5,10–CH=THF by 19.62%, 23.68%, and 39.05% in 2020, and 46.71%, 23.65%, and 71.54% in 2021, respectively, induced by N stress, were observed as well (Figure 3). Further, the negative effects of low-N stress, in 2021, on folate derivatives contents were much more obvious than those in 2020. Nitrogen deficiency did not significantly affect the content of DHF, but the content of FA enhanced by 168.54% in 2020. These two folate derivatives were in the range of < 1.5 μg per 100 g grains (Figure 3). The study to evaluate the effect of N regimes on folate level in cereals is sanity. Thus, the effect of N deficiency on grain folate derivatives in this study can provide more precise information regarding how to modulate natural folate components by agronomic fertilization measures.

**Figure 4:**
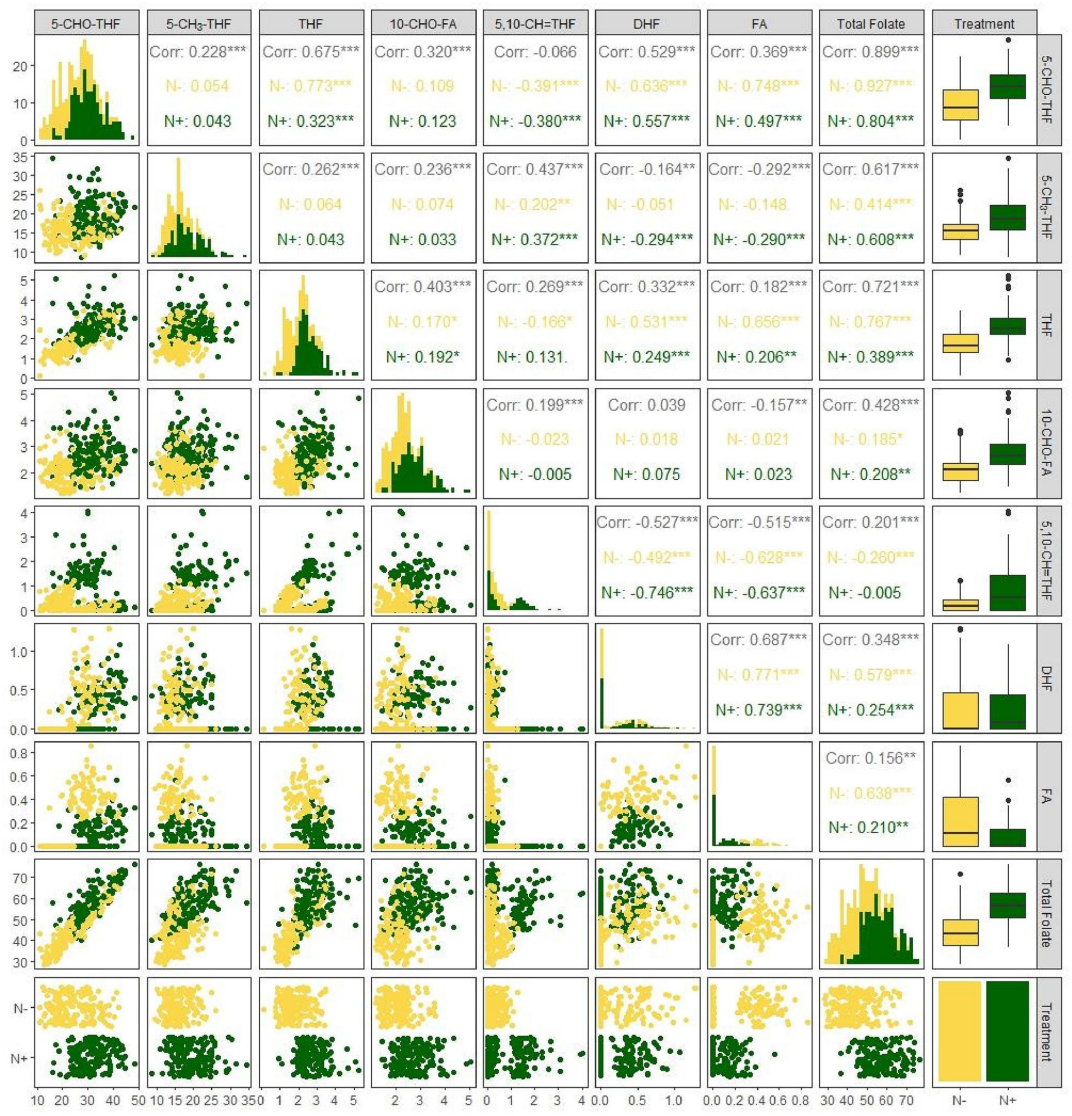
Correlation among the content of folate derivatives 5–CHO–THF, 5–CH_3_-THF, THF, 10–CHO–FA, 5,10–CH=THF, DHF, FA, and total folate grown with or without N fertilization. N–, N deficiency; N+, N applied treatment. **, *** Indicate significant differences at 0.01 and 0.001.

The obvious effects of N deficiency on the correlation between each folate derivative content and total folate content were not noticed, usually. All folate derivatives were positively associated with total folate content with the exception of 5,10–CH=THF for both N regimes. Among these seven folate derivatives, the highest correlation was observed between 5–CHO–THF and total folate (*r* = 0.804^***^ with N; *r* = 0.927^***^ without N *r* = 0.90^***^), which may have been caused by its high abundance in foxtail millet. The 5,10–CH=THF and total folate content had a negative correlation (*r* = −0.260^***^) under N deficiency, while had no correlation when N was supplied (Figure 4). Consistent with the previous study (7), presently a negative correlation (*r* = −0.380^***^ with N; r = –0.391^***^ without N) was also observed between 5–CHO–THF and 5,10- CH=THF (Figure 4), which may result from the interconversion relationship between these two components (23).

### 3.3. Effect of N deficiency on crop production and nutrient accumulation in foxtail millet

**Figure 5:**
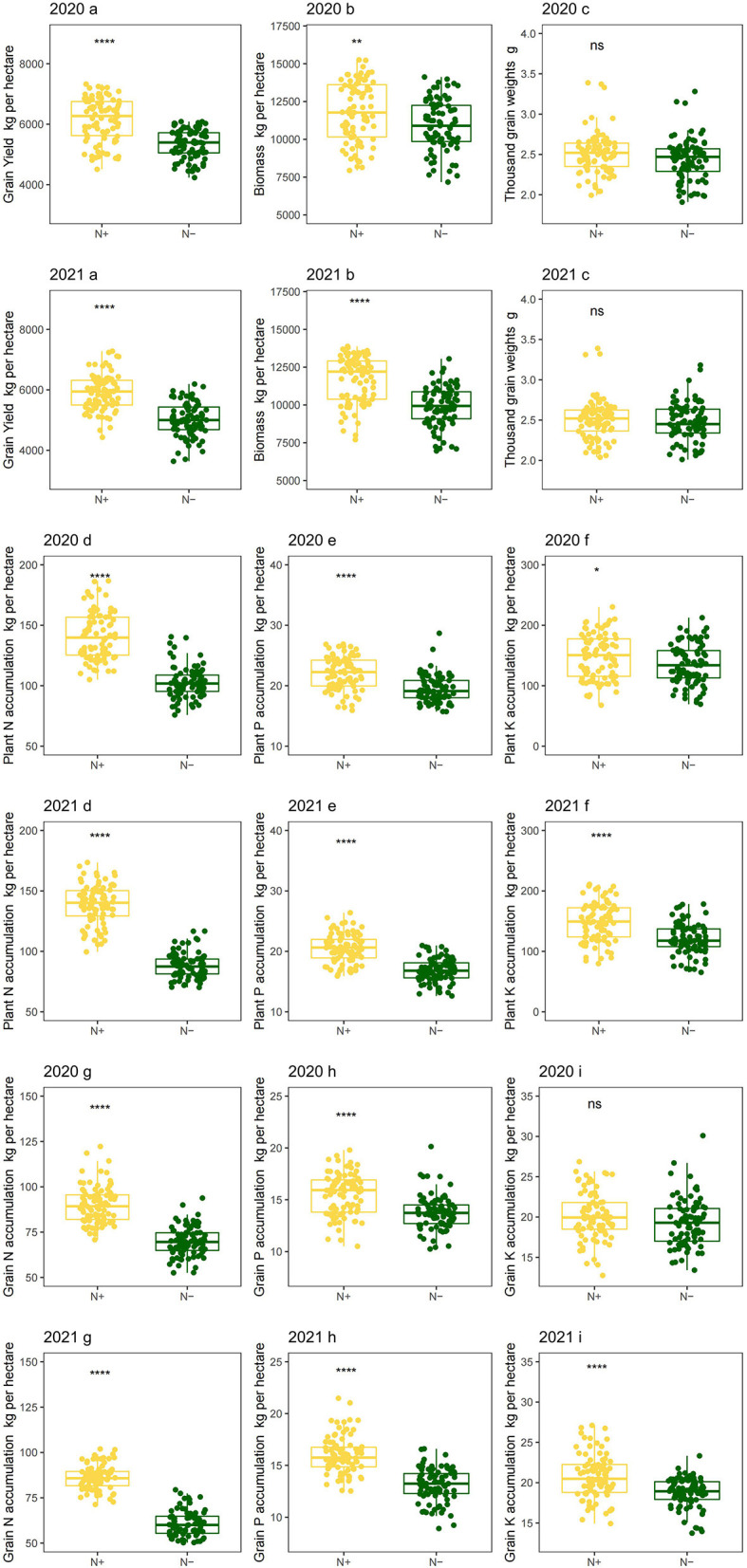
Crop production and nutrient accumulation of foxtail millet under low-N stress in 2 years. Grain yield per hectare (2020a in 2020; 2021a in 2021), aboveground biomass per hectare (2020b in 2020; 2021b in 2021), thousand grain weights (2020c in 2020; 2021c in 2021), plant N accumulation per hectare (2020d in 2020; 2021d in 2021), plant P accumulation per hectare (2020e in 2020; 2021e in 2021), plant K accumulation per hectare (2020f in 2020; 2021f in 2021), grain N accumulation per hectare (2020g in 2020; 2021g in 2021), grain P accumulation per hectare (2020h in 2020; 2021h in 2021) and grain K accumulation per hectare (2020i in 2020; 2021i in 2021). N–, N deficiency; N+, N applied treatment. *, **, **** and ns indicate significant differences at 0.05, 0.01, 0.0001 and no significant difference, respectively. Error bars represent the standard error of folate derivatives.

Low N stress had negative effects on grain yield and aboveground biomass. In compared with the N application, the average grain yield decreased by 13.24% and 15.03% in 2020 and 2021, respectively (Figure 5 2020a and 2021a), and biomass decreased by 7.58% and 14.78% in 2020 and 2021, respectively (Figure 5 2020b and 2021b), which indicated that the grain yield and aboveground biomass are known to be directly associated with N supply. Lower biomass and grain yield, induced by N deficiency, were observed in the present and other studies (42). The effects of N deficiency on grain weight are conflicting. Low N could lead to increases in potential grain weight for wheat due to grain abortion (43). However, it is also found that N deficiency also had negative effects on wheat grain weight due to reductions in source strength (44). Whereas, N application had no significant effects on thousand-grain weights in this study of foxtail millet (Figure 5 2020c and 2021c). This probably could have led to a balance between these two effects under N deficient conditions.

The plant and grain nutrient accumulation were strongly limited by N deficiency. Low-N induced clearly decreases in N accumulation by 27.14% and 36.62% on average in 2020 and 2021, respectively, for the whole plant (Figure 5 2020d and 2021d). Accordingly, N accumulation decreased by 22.36% and 30.71% for grains in 2020 and 2021, respectively (Figure 5 2020g and 2021g). This finding was consistent with other studies in foxtail millet (45). Consistently, a similar trend was also observed in the phosphorus (P) and potassium (K) accumulation for whole plant and grain tissues for 2 years, although there was no significant difference in grain K accumulation between two N treatments in 2020 (Figure 5 2020i). Perhaps it is due to the low proportion for K of crop reproductive organs (46). Moreover, N stress, which was not serious in 2020, did diminish K accumulation in grain. These negative effects might be due to decreased P and K absorption by N deficiency in foxtail millets (47).

Applied N can increase N accumulation at the whole-plant level (48), which is in line with the present results. Nitrogen, helpful for a relative massive root system development, improves P and K accumulation in plants. The enhanced P and K accumulation might be associated with increased biomass and yield.

### 3.4. Association of foxtail millet folate contents with grain weight and nutrient accumulation

**Figure 6:**
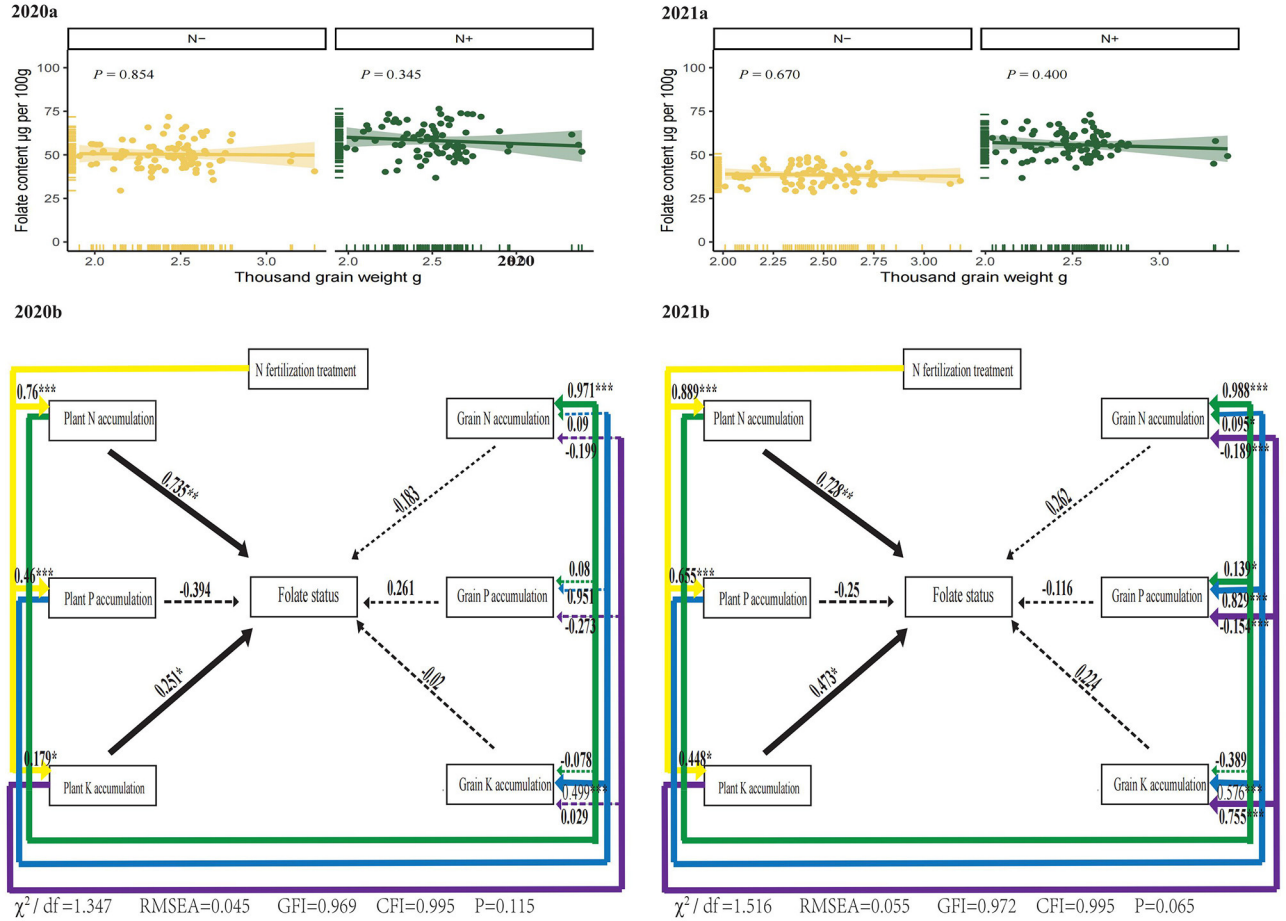
Correlation of foxtail millet thousand grain weights with total folate in 2 years. Structural equation models (SEM) to evaluate the direct and indirect effects of plant nutrient accumulation, grain nutrient accumulation, above ground biomass and grain yield on folate levels in foxtail millet. N–, N deficiency; N+, N applied treatment. The goodness-of-fit index (GFI), Bentler comparative fit index (CFI), Chi-square test and root mean square error of approximation (RMSEA) indicate the goodness-of-fit of the models to the original data. * and *** indicate the standard of significance at 0.05 and 0.001 level, respectively.

According to Giordano et al. (49), folates were unevenly distributed in wheat grains, and wheat germs had a higher concentration of folates than their outer layer. Usually, thousand-grain weight and folate content had a negative correlation in Wheat (7, 9). However, in the present study, there was no statistically significant relationship appeared between thousand-grain weights and total folate in 2 years under two N treatments (Figure 6 2020 and 2021). The effect of seed morphological traits on foxtail millet folates is unknown. It is noticeable that the embryo of millet represented a larger proportion of the grain weight than in other cereal grains (50). And the foxtail millet grains are much smaller than other cereals. The wheat thousand-grain weight, for example, is about 20-fold higher than that of foxtail millet. Therefore, the effects of dilution on folates in foxtail millet were much lower than that of wheat, which probably supported no significant relationship appeared between thousand grain weights and folates in foxtail millet.

Structural equation models (SEMs) are statistical procedures for testing measurement, functional, predictive, and causal hypotheses. We further constructed SEMs to explore the direct and indirect relationships among N Fertilization, plant nutrient uptake, grain nutrient uptake and folate levels in foxtail millets (Figure 6), in terms of the effects of N deficiency on nutrient accumulation described in Figure 5. Applied N had a strong positive and indirect effect on the foxtail millet folate through positively influencing plant N accumulation and plant K accumulation in 2 years (Figure 6). The interactions between folate levels in foxtail millets (a latent variable) and plant N accumulation or plant K accumulation (observed variables) revealed that plant N and K accumulation affected folate levels more obviously than grains. Plant N and K status, which were influenced by N regimes, had a strong positive effect on folates.

## 4. Conclusion

We used HPLC-MS/MS techniques to investigate the folate derivatives of 29 different foxtail millet cultivars for 2 years under two N treatment rates (0 and 150 kg N ha^−1^). With the application of N, the cultivar Jingu21 recorded the highest mean folate content among the 29 foxtail millet cultivars (71.03 μg per 100 g of grains) for 2 years. Whereas folate contents of Jigu22 and Longgu25 were < 50 μg per 100 g grains. The folate content for remaining foxtail millet cultivars ranged from 50 to 70 μg per 100 g grains. The contents of total folate and derivatives in foxtail millet were significantly reduced by N deficiency. The effects of N regimes on folate contents were much more evident compared with the impacts induced by cultivars. SEMs demonstrated N fertilization affected folate content of foxtail millet positively, brought about by enhanced N and K accumulation in plant aboveground. Furthermore, foxtail millet cultivars with higher level folate might have the ability to accumulate more N at the same N application rates. Folate content of high folate enriched cultivars was prone to be reduced by N deficiency, which indicated that much attention should be paid to N management when elite folate cultivars were cultivated, especially in infertile soil conditions, to ensure foxtail millet grain quality.

## Chemical compounds studied in this article

5-Formyl-tetrahydrofolate (PubChem CID: 135403648); 5-Methyl-tetrahydrofolate (PubChem CID: 135483998); Tetrahydrofolate (PubChem CID: 135444742); 10-Formyl-folic acid (PubChem CID: 135405023); 5,10-Methenyl-tetrahydrofolate (PubChem CID: 135398657); Dihydrofolate (PubChem CID: 135398604); Folic acid (PubChem CID: 135398658); Methotrexate (PubChem CID: 126941); Sodium phosphate monobasic (PubChem CID: 23672064); Sodium phosphate dibasic (PubChem CID: 24203); Sodium ascorbate (PubChem CID: 23667548); β-Mercaptoethanol (PubChem CID: 1567); Acetonitrile (PubChem CID: 6342); Formic acid (PubChem CID: 284).

## Data availability statement

The data that support the findings of this study are available from the corresponding author, X-yJ, upon reasonable request.

## Author contributions

YW: data curation, formal analysis, investigation, methodology, and writing-original draft. J-sW: supervision and validation. E-wD: validation. Q-xL: visualization. L-gW: project administration. E-yC: investigation and resources. X-yJ and X-mD: conceptualization, funding acquisition, project administration, resources, and writing—review and editing. All authors contributed to the article and approved the submitted version.
