# Supplementary figures and images for: Foxtail millet [Setaria italica (L.) P. Beauv.] grown under nitrogen deficiency exhibits a lower folate contents
Source: Front Nutr. 2023 Jan 18;10:1035739. doi: 10.3389/fnut.2023.1035739 (PMC9889834; doi:10.3389/fnut.2023.1035739)

2020 a Jingu21 N-

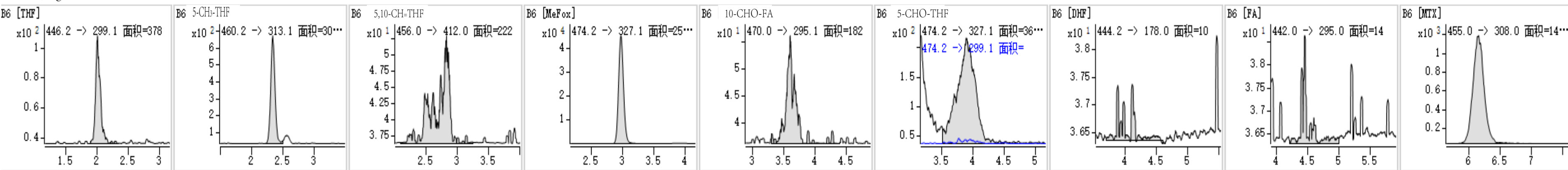

2020 a Jingu21 N+

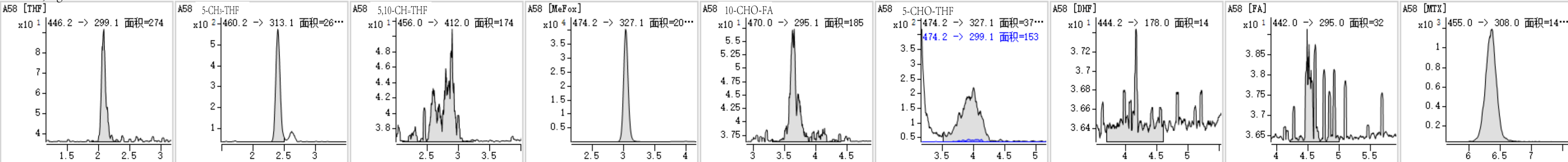

2021 a Jingu21 N-

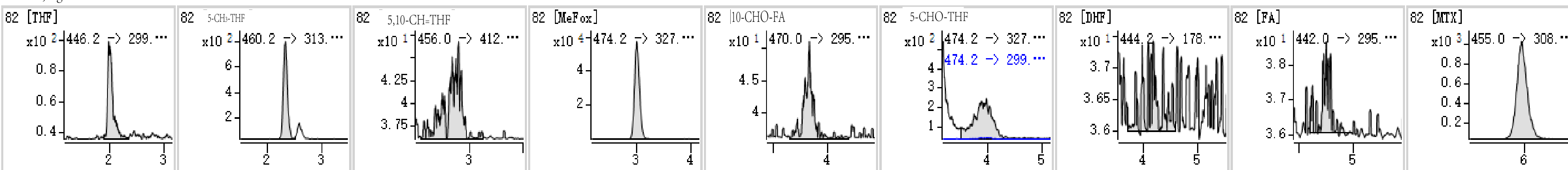

2021 a Jingu21 N+

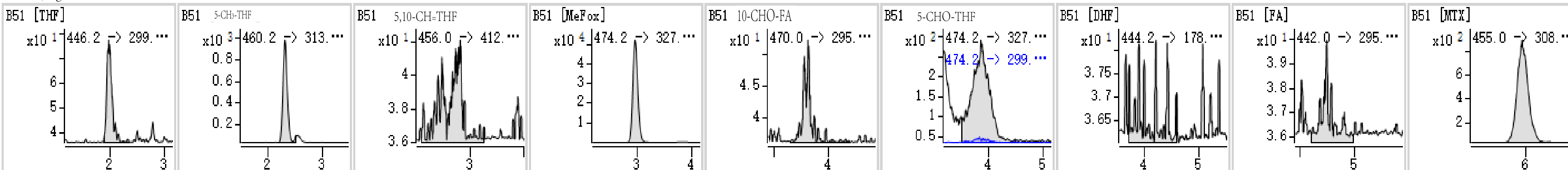

Supplement: Supplementary file 4 [file Data_Sheet_1.PDF]
